# Therapeutic effect of Micro-Implant Anchorage in orthodontics

**DOI:** 10.12669/pjms.40.10.10442

**Published:** 2024-11

**Authors:** Yarong Shi, Lei Mi, Jie Liu, Xiaoyao Liu, Yanan Hao

**Affiliations:** 1 Yarong Shi, Department of Stomatology, Yulin First Hospital, Yulin, Shaanxi Province 719000, P.R. China; 2 Lei Mi, Department of Stomatology, Yulin First Hospital, Yulin, Shaanxi Province 719000, P.R. China; 3 Jie Liu Department of Stomatology, The Second Affiliated Hospital of Harbin Medical University, Harbin, Heilongjiang Province 150086, P.R. China; 4 Xiaoyao Liu Department of Stomatology, The First Affiliated Hospital of Harbin Medical University, Harbin, Heilongjiang Province 150001, P.R. China; 5 Yanan Hao Department of Stomatology, Yulin First Hospital, Yulin, Shaanxi Province 719000, P.R. China

**Keywords:** Micro implant anchorage, Straight line appliance, Orthodontic

## Abstract

**Objective:**

Micro implant anchorage (MIA), as a new orthodontic treatment technique, has the characteristics of simple operation, light trauma, and high stability, and has been widely used in clinical practice. This study compared the therapeutic effects of MIA and the traditional straight wire appliance (SWA) in orthodontic practice.

**Methods:**

Clinical data of 119 patients who underwent orthodontic treatment in Yulin First Hospital from January 2022 to January 2023 were retrospectively analyzed. According to the treatment records, 61 patients received MIA orthodontic treatment (MIA group), and 58 patients received traditional straight wire appliance (SWA) orthodontic treatment (SWA group). Treatment effect, periodontal index, and levels of osteoprotegerin (OPG), interleukin-6 (IL6) and high-sensitivity C-reactive protein (hsCRP) of the two groups were compared.

**Results:**

The total efficacy of MIA group was significantly higher than that of SWA group (*P*<0.05). After the treatment, bleeding index (BI), plaque index (PLI), probing depth (PD) and looseness in the MIA group were significantly lower than those in the SWA group (*P*<0.05). After the treatment, levels of OPG were significantly higher, while IL6 and hsCRP levels were significantly lower in the MIA compared to the SWA group (*P*<0.05). MIA was associated with significantly lower complication rate compared to the SWA (*P*<0.05).

**Conclusions:**

Compared with SWA, MIA is associated with better orthodontic effect. This mode of treatment can increase OPG levels, reduce inflammation, and lower the risk of complications.

## INTRODUCTION

Orthodontics refers to the treatment process that aims to correct misaligned teeth, and abnormal tooth shape and color.^1^-^3^ Traditional straight wire appliance (SWA) for fixed orthodontic treatment have become an important measure due to its firmness, stability, and high movement accuracy.^4^,^5^ However, SWA is associated with some disadvantages such as poor aesthetics, long treatment time, and easy detachment of brackets.^5^,^6^ Micro-implant anchorage (MIA) recently emerged as an alternative to SWA, and is gradually being applied in clinical practice. Compared with traditional orthodontic methods, MIA is simpler to perform, does not require fixed devices, and can be easily removed, solving problems such as small gaps and unsuitable teeth brackets.^7^ However, data on the efficiency of MIA compared to the traditional SWA are scarce.

Mechanical stimulation during orthodontic treatment is accompanied by bone remodeling,^8^ and activation of inflammatory reactions that may affect the health of periodontal tissue.^9^,^10^ Studies show that mechanical strain is associated with the regulation of the osteoprotegerin (OPG), a decoy receptor of the receptor activator of nuclear factor-кB ligand (RANKL). OPG binding to RANKL have been shown to play important roles in bone remodeling by reducing osteoclast production.^10^ In orthodontic treatment, OPG may affect the remodeling of alveolar bone, thereby affecting tooth movement and orthodontic effect.^6^,^10^

Additionally, activation of inflammatory processes and the release of pro-inflammatory cytokines as a response to mechanic stimulation is considered the main reason for the failure of orthodontic treatment.^8^-^10^ The levels of IL-6 and hs CRP can reflect the severity of periodontitis.^9^,^10^ SWA has a complex structure and complicated operation, which can easily cause damage to the dental and periodontal tissues, leading to inflammatory reactions. However, MIA has advantages such as small size, easy operation and use, and minimal trauma. Therefore, this study used SWA as the control group to explore the therapeutic effect of MIA in orthodontics, as well as its impact on periodontal index, osteoprotegerin, and inflammatory response intended to provide reference for clinical treatment.

## METHODS

Clinical records of patients, undergoing orthodontic treatment in Yulin First Hospital from January 2022 to January 2023, were retrospectively collected. According to the method of orthodontic treatment, patients who received MIA orthodontic treatment were included in the MIA group, and patients who received SWA were included in the SWA group.

### Ethical Approval:

The ethics committee of our hospital approved this retrospective study with the number: 2023-002, Date: October 30, 2023.

### Inclusion criteria:

- Meet the criteria for orthodontic treatment.^1^
- Age ≥ 18 years.
- Complete clinical data.

### Exclusion criteria:

- Patients with oral diseases (periodontitis, etc.).
- Patients with severe mental disorders.
- Pregnant or lactating women.
- Patients with systemic diseases and coagulation disorders.

### Straight Wire Appliance (SWA):

For the procedure, 0.02% chlorhexidine mouthwash and 2% lidocaine were used for local anesthesia. Soft stainless-steel tie wire with a diameter of 0.2-0.5mm was used to fix the arch wire. First, the ligation wire was placed into the wing groove of the bracket and tightened. Ligation wire was then rotated and the knot tied. At the same time, the arch wire was pressed into the groove of the bracket. Finally, the excessively long ligature wire was cut off and the end was pressed towards the inside of the arch wire to prevent the ligature wire from puncturing the patient’s oral mucosa. The initial traction force was about 300g per side, and follow-up visits were conducted once a month to adjust the traction force based on the patient’s dental recovery. If necessary, the length of the bow wire was further supplemented, and the traction force was adjusted.

### Micro-implant anchorage (MIA):

Local moist anesthesia on the oral cavity was initiated, and the location of implantation inside the oral cavity was marked. Copper wire was used to separate the upcoming micro-implants to avoid obstacles during implantation. Before implanting self-tapping micro titanium nail anchorage, the patient’s tooth root morphology and surrounding tissue were checked to confirm that there were no abnormalities. The upper jaw model of self-tapping micro titanium nail support was CBMA1.5-11, and the lower jaw model was CBMA1.5-9, selected according to the individual patient’s situation. During the surgery, a surgical knife was used to cut open the mucosa of the patient’s alveolar flap to avoid damaging the alveolar bone. Finally, X-ray was used to confirm the relationship between the tooth tip and root, ensuring the correct placement of the implant. Follow up visits were conducted once a month to replace the chain rubber band, and adjust and supplement the status of the implantation site.

### Data collection:

- Patient baseline data, including gender, age, type of deformity and BMI.
- Therapeutic effects.
- Periodontal index, including bleeding indicators, looseness, plaque index, and periodontal pocket depth.
- Biochemical indicators, including OPG, interleukin-6 (IL6), and high-sensitivity C-reactive protein (hsCRP) levels.
- The incidence of complications, including pain, soft tissue swelling, oral infection and oral inflammation.

### Treatment effectiveness:

- Significant effectiveness- patient’s teeth are arranged neatly, the relationship between molars and cusps is normal, the anterior teeth are covered normally, and the facial shape is significantly improved.
- Effective- patient’s teeth are arranged neatly, the relationship between molars and cusps is improved, the anterior teeth are covered normally, and the facial shape improved.
- Invalid- patient’s teeth are arranged neatly, and there is no significant improvement in the facial shape. The anterior teeth overlap is basically normal.
- Total effective rate = (significant+effective)/total number of cases × 100%.

### Periodontal index evaluation:

### Bleeding index (BI):

Periodontal probes are used for bleeding on probing test. Based on the number and severity of blood stains, BI was classified into levels 0-5. Level 0: No bleeding; *Level 1:* Minor bleeding with small blood spots on the probe; *Level 2:* Moderate bleeding with obvious blood stains on the probe; *Level 3:* Massive bleeding, blood flowing down the edge of the gums; *Level 4:* Spontaneous bleeding with red or purple gums, bleeding occurs when there is no stimulation; Level 5: Spontaneous bleeding with deep red or purple gums and ulceration.^11^

### Looseness was classified as follows:

0 points (no looseness)- looseness amplitude of teeth in the inner and outer directions is less than 1mm, and slight looseness of the front and back tongue lips; Two points- the degree of looseness of teeth in both internal and external directions is 1-2mm, or there is looseness in both internal and external directions, with obvious looseness towards the tongue and lip; Three points- looseness of teeth in the inner and outer directions is greater than 2mm, or if there is looseness in the labial and lingual sides, mesial and distal directions.^12^

Plaque index (PLI) determined by visual examination and by lightly scratching the tooth surface with a probe, and scored based on the amount and thickness of plaque. Four tooth surfaces (mesial buccal surface, median buccal surface, distal buccal surface, and lingual surface) per tooth are examined; The score for each tooth is the sum of the four tooth surface scores divided by four, and the personal score is the sum of the scores for each tooth divided by the number of teeth being tested. The scoring criteria for plaque index were as follows: 0 points=no bacterial plaque in the gingival margin area; 1 point=the tooth surface divided into gingival margin area has plaque that is not visible to the naked eye, but plaque can be observed on the side of the probe tip; 2 points=moderate amount of plaque visible at the gingival margin or adjacent surface; 3 points=There is a large amount of soft dirt in the gingival sulcus or gingival margin area, that is, the adjacent surface.^13^

Probe depth (PD) refers to the depth at which a probe is inserted into the gingival sulcus along the long axis of the tooth. According to the severity of the periodontal pocket, the depth of the periodontal pocket was divided into the following three levels: 1 point-the probing depth of the periodontal pocket is less than 4mm, and the oral CT shows that the absorption length of the alveolar bone is within one-third of the range of the tooth root; 2 points-the probing depth of the periodontal pocket is less than 6mm, and the oral CT shows that the absorption length of the alveolar bone is between half and one-third of the root of the tooth, the probe slightly touches the tooth, slight looseness of the tooth; 3 points-the probing depth of the periodontal pocket is greater than 6mm, and the oral CT shows that the absorption length of the alveolar bone exceeds half of the root, the probe slightly touches the teeth and the teeth become loose.^11^,^13^

### Biochemical indicator:

Briefly, oral cavity was rinsed with physiological saline to remove oral debris. Gingival crevicular fluid (GCF) was collected from periodontal tissue and gingival crevicular floor using filter paper strips. Inflammatory factors in the supernatant of the patient’s GCF, including IL6 and hsCRP, were measured by enzyme-linked immunosorbent assay. Real time PCR was used to detect the OPG content in osteoclasts.

### Statistical analysis:

Data were analyzed using SPSS version 26.0 (IBM Corp, Armonk, NY, USA). For categorical variables, frequency distribution was expressed as a percentage. The chi square test was used to compare categorical variables between two groups, such as gender distribution, types of dental deformities, and the presence of complications. The measurement data were represented as mean ± standard deviation, independent sample *t*-test was used for inter group comparison, and paired *t*-test was used for intra group before and after treatment. *P*-value less than 0.05 was considered an indicator of statistically significant differences.

## RESULTS

Clinical data of 119 patients (57 males and 62 females) who underwent orthodontic treatment were analyzed. Age of the patients ranged from 18 to 44 years, with a mean of 30.04 ± 6.63 years; 58 patients were included in the SWA group and 61 were included in the MIA group. There was no statistically significant difference in the baseline data between the two groups of patients (*P*>0.05). Table-I. The total effective rate of treatment in the MIA group was significantly higher than that in the SWA group (*P*<0.05). Table-II

**Table-I:** Comparison of baseline data between two groups.

Baseline data	SWA group (n=58)	MIA group (n=61)	χ^2^/t	P
Gender (male/female)	25/33	32/29	1.043	0.307
Age (year)	29.05±6.81	30.98±6.36	-1.600	0.112
Types of dental deformities			2.496	0.476
Malalignment	9 (15.52)	14 (22.95)		
Maxillary protrusion	24 (41.38)	18 (29.51)		
Chin retraction	19 (32.76)	20 (32.79)		
supernumerary teeth	6 (10.34)	9 (14.75)		
BMI (kg/m^2^)	22.92±2.49	23.40±2.60	-1.030	0.305

**Table-II:** Comparison of treatment effects between two groups.

Group	Significant effect	Effective	Invalid	Total effective rate (%)
SWA group (n=58)	30 (51.72)	17 (29.31)	11 (18.97)	47 (81.03)
MIA group (n=61)	39 (63.93)	18 (29.51)	4 (6.56)	57 (93.44)
*χ^2^*				4.155
*P*				0.042

**Fig.1:**
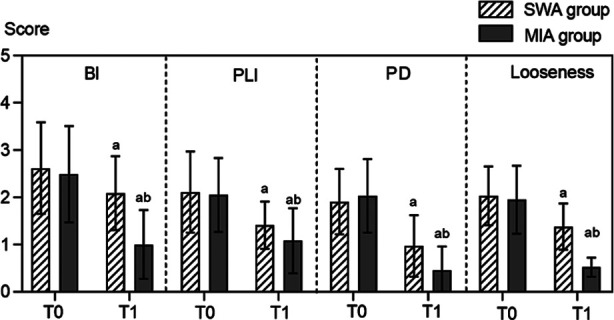
Comparison of periodontal indices between two groups; SWA: straight-wire appliance; MIA: Micro-implant anchorage; BI: Bleeding Index; Plaque Index; PD: Probing depth; T0: Before treatment; T1: After treatment.

Before the treatment, there was no statistically significant difference in BI, PLI, PD, and looseness between the two groups (*P*>0.05). After the treatment, BI, PLI, PD, and looseness of both groups significantly decreased, and was markedly lower in the MIA group compared to the SWA group (*P*<0.05). Fig.1

**Fig.2:**
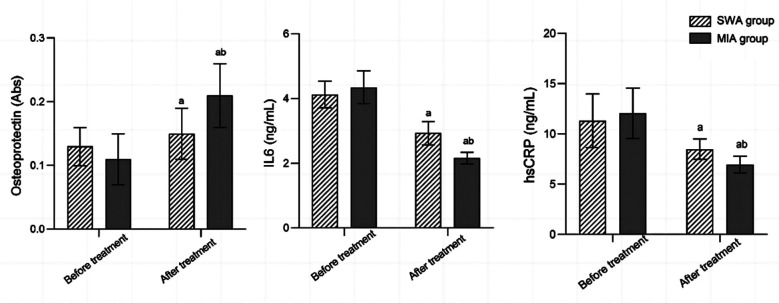
Comparison of levels of OPG, IL6, and hsCRP between two groups; OPG: osteoprotegerin; IL6: Interleukin-6; hS-CRP: high sensitivity C-reactive protein (hsCRP); SWA: straight-wire appliance; MIA: Micro-implant anchorage.

Before the treatment, levels of OPG, IL6, and hsCRP were similar in the two groups (*P*>0.05). After the treatment, levels of OPG in both groups were significantly higher than before the treatment, and significantly higher in the MIA group compared to the SWA group. On the other hand, post-treatment IL6 and hsCRP levels were significantly lower in both groups, and markedly lower in the MIA group than in the SWA group (*P*<0.05). Fig.2. The total incidence of complications in the MIA group was significantly lower than that in the SWA group (*P*<0.05). Table-III

**Table-III:** Complications of the two groups.

Group	Pain	Soft tissue edema	Oral infection	Oral inflammation	Total incidence rate (%)
SWA group (n=58)	5 (8.62)	1 (1.72)	2 (3.45)	2 (3.45)	10 (17.24)
MIA group (n=61)	1 (1.64)	0 (0.00)	1 (1.64)	1 (1.64)	3 (4.92)
*χ^2^*					4.640
*P*					0.031

## DISCUSSION

The results of this study show that MIA orthodontics is associated with significant improvement in periodontal indexes and inflammatory status of the patients compared to the SWA. In the past few years, MIA has become increasingly popular in the clinical management of orthodontic patients. Introducing temporary anchoring devices makes it possible to overcome traditional anchoring and its limitations. Zhang et al.^14^ found that compared to traditional sliding linear appliances, using the MIA orthodontic system can more effectively rotate the functional occlusal plane counterclockwise and reduce the mandibular plane angle.

A meta-analysis by Wang K et al.^15^ also demonstrated that the clinical application of MIA in the treatment of angle II malocclusion provides reliable evidence-based benefits. A study by Lin G et al.^16^ showed that MIA combined with transparent orthodontic appliances can better protect the anchorage of the mandibular central incisors and improve the efficiency of distal molar crown. These results are in agreement with the outcomes of our study.

Our results indicate that MIA is associated with significantly better periodontal index compared to SWA. Patients in the MIA group had a lower degree of gingival inflammation and periodontal infection, thus enhancing tooth stability. In the study comparing the orthodontic effects of MIA and traditional orthodontic methods on young orthodontic patients, Wang T et al.^17^ showed that MIA treatment resulted in better PI, and lower incidence of adverse reactions compared to the traditional orthodontic methods. This is also consistent with our results.

In this study, the levels of OPG in the MIA group were higher, while the levels of IL6 and hsCRP were lower than those in the SWA group, indicating that MIA treatment has a positive effect on the protection and inflammation control of orthodontic bone tissue.^18^ MIA can promote the formation of fibrotic tissue between bones and improve implant stability during treatment.^18^,^19^ It does not require opening the gingival tissue, but can be removed by reverse rotation, resulting in less trauma and milder periodontitis reactions.^18^-^20^ OPG is an important bone metabolism regulatory factor that can inhibit osteoclast activity, promote osteoblast formation, and help protect bone tissue.^18^ Our results suggest that MIA treatment may stimulate protection and regeneration of bone tissue in orthodontic patients.^21^,^22^

Patients in the MIA group in our study had significantly lower incidence of adverse effects than the SWA group. The process of orthodontic treatment is often accompanied by pain, since the applied force affects peripheral nerve fibers and terminals in periodontal tissue.^23^,^24^ During the process of orthodontic treatment, due to the force on the teeth, it may cause dental acid and pain.^25^ Orthodontic problems may also lead to permanent damage to periodontal tissue, and incorrect orthodontic force can disrupt the activity of osteoblasts and osteoclasts around the alveolar bone, which can be absorbed by the tooth root.^25^,^26^ Our study indicates that compared to traditional orthodontic methods, MIA is associated with significantly fewer complications in oral orthodontics. We may speculate that MIA method is easy and minimally invasive, is associated with high material strength and corrosion resistance, is suitable for various types of malocclusions, and leads to minimal trauma during implantation and removal.^27^,^28^

### Limitations:

Firstly, this is a single center retrospective study. Strict inclusion and exclusion criteria and limited sample size may result in some bias. Secondly, no analysis has been conducted for specific populations, such as teenagers. Thirdly, the follow-up time was relatively short, and the long-term efficacy of MIA orthodontic treatment on patients was not observed. Finally, the impact of the two methods on the long-term functional recovery of patients was not analyzed. Further multi-center studies with larger sample size and extended follow-up times are needed to further validate the conclusions of our study.

## CONCLUSION

MIA is associated with better orthodontic effect compared to SWA, and can increase OPG levels, reduce inflammation, and lower the risk of complications.

### Authors’ contributions:

**YS:** Conceived and designed the study.

**LM**, **JL**, **XL** and **YH:** Collected the data, performed the analysis and Review

**YS:** Was involved in the writing of the manuscript and is responsible for the integrity of the study.

All authors have read and approved the final manuscript.
